# Elevated Phosphorylated Tau and Amyloid‐Beta 42 in Sleep Apnoea: Evidence From a Systematic Review and Meta‐Analysis

**DOI:** 10.1155/bmri/4366369

**Published:** 2026-11-15

**Authors:** Sara Parsons, Sam Williamson, Sonia A. L. Corrêa

**Affiliations:** ^1^ Respiratory Physiology St George′s University Hospitals NHS Foundation Trust London UK; ^2^ Faculty of Science and Engineering Manchester Metropolitan University Manchester UK mmu.ac.uk

**Keywords:** Alzheimer′s disease, hypoxia, obstructive sleep apnoea, oxygen desaturation index, plasma biomarkers

## Abstract

**Background:**

Alzheimer′s disease (AD) biomarkers were detected in the cerebrospinal fluid, plasma and brain of obstructive sleep apnoea hypopnoea syndrome (OSAHS) sufferers. The mechanism mediating these changes remains disputed. This study aims to investigate the relationship between chronic intermittent hypoxia caused by OSAHS and AD blood biomarker levels, allowing a better understanding and consideration of the systemic impact of OSAHS on AD development.

**Methods:**

We searched PubMed, Scopus and Web of Science databases and conducted the research according the PRISMA 2020 guidelines. Studies were included if they reported hypoxic parameters in an OASHS group and a control group and analyses of blood biomarker levels including amyloid‐beta and tau. The Newcastle–Ottawa Scale was used to assess study quality.

**Results:**

Eight studies including 435 OSAHS patients and 281 controls were assessed. Pooled analysis showed elevated A*β*_42_ (SMD = 0.27, 95*%*(CI) = 0.03 to 0.51, *I* ^2^ = 56*%*, *p* = 0.03), T‐tau (SMD = 0.60, 95*%*(CI) = 0.26 *to* 0.94, *I* ^2^ = 63*%*, *p* = 0.0006) and p‐tau181 (SMD = 0.41, 95*%*(CI) = 0.03 to 0.80, effect size = 2.12, *I* ^2^ = 51*%*, *p* = 0.03) in the OSAHS group compared with controls, though no difference in A*β*_40_ (SMD = 0.33, 95*%*(CI) = −0.27 to 0.94, effect size = 1.09, *I* ^2^ = 77*%*, *p* = 0.28) levels was observed. Reduced mean oxygen saturation is associated with elevated T‐tau (SMD = −0.36, 95*%*(CI) = −0.62 to − 0.10, *I* ^2^ = 0*%*, *p* = 0.007) and p‐tau181 (SMD = −0.61, 95*%*(CI) = −0.96 to − 0.27, *I* ^2^ = 0*%*, *p* = 0.0004) but not with A*β*_42_ (SMD = −0.19, 95*%*(CI) = −0.70 to 0.32, *I* ^2^ = 76*%*, *p* = 0.46). Elevated oxygen desaturation index was associated with increased A*β*_42_ (SMD = −0.37, 95*%*(CI) = −0.56 to − 0.18, *I* ^2^ = 49*%*, *p* = 0.0001) and T‐tau (SMD = −0.54, 95*%*(CI) = −0.90 to − 0.17, *I* ^2^ = 58*%*, *p* = 0.004).

**Conclusions:**

Findings show that chronic intermittent hypoxia is associated with increased blood A*β*_42_ levels,_,_ but not A*β*_40_, in the OSAHS group. This may suggest that hypoxia contributes to processes regulating the production and degradation of neurotoxic amyloid‐beta peptides. Further experimental investigation is required to determine whether treating OSAHS and restoring optimal oxygen saturation conditions in the brain affects blood A*β*_42_ levels and whether OSAHS is a modifiable disorder that may increase the risk of developing AD.

## 1. Introduction

Obstructive sleep apnoea hypopnoea syndrome (OSAHS) is a sleep breathing disorder that causes upper airway collapse leading to complete or partial obstruction of breathing [1]. The repetitive airway collapses throughout the night cause interruption in breathing and intermittent reductions in oxygen levels in the blood. Frequent decreases and increases in blood oxygen saturation and and the resulting intermittent hypoxia (IH) negatively affect brain function and structure. As OSAHS is often unknown to the person and consequently is commonly left undiagnosed for years, these individuals suffer from chronic intermittent hypoxia (CIH) [2]. CIH has been proposed to contribute to the decline in cognitive function observed in those with OSAHS [1, 3]. Over time, the intermittent lack of oxygen in the brain may contribute to the detection of Alzheimer′s disease (AD) biological biomarkers in OSAHS sufferers [4, 5].

AD is the most common form of dementia affecting 50 million people worldwide [6]. The prevalence of dementia in the UK is estimated to have a 75% increase with a population growing from 907,331 in 2019 to 1,592,475 by 2050 [7]. With no preventative or effective treatment strategies currently available, AD is a great social, economic and health burden. Risk factors have been identified to include age and sex; however, these are unmodifiable [8]. Subsequently, there has been an increased interest to focus on modifiable risk factors such as diabetes, hypertension and social isolation [9], and recent evidence suggests OSAHS as a potential risk factor for AD [10, 11]. Although the mechanisms associating OSAHS and AD are not established, it is important to understand the influence of OSAHS on the development of AD pathology [5, 11].

The pathological hallmark of AD is the accumulation and aggregation of soluble extracellular amyloid‐beta (A*β*) oligomers with consequent formation of insoluble amyloid plaques and intracellular neurofibrillary tangles formed from hyperphosphorylated tau protein [12, 13]. Excessive accumulation of these proteins in the brain disrupts neuronal communication and function, leading to neuronal death in areas of the brain involved in learning and memory and therefore contributes to cognitive impairment [14, 15]. Evaluation of concentration changes of these proteins in the cerebrospinal fluid (CSF) and blood has been used in the diagnosis of AD [16]. Changes in AD biomarkers have been detected in the CSF [17–19] and blood [20–22] of individuals with OSAHS, suggesting that these individuals have biological changes associated with AD pathology [16]. However, the mechanism underlying these early changes in AD‐related proteins in those with OSAHS is not established. CIH may provide a mechanism for changes in A*β* levels. It has been hypothesised that hypoxia regulates amyloid precursor protein (APP) processing and production of A*β* through the amyloidogenic pathway [12]. Experimental studies showed that IH increases hypoxia‐inducible factor 1*α* (HIF‐1*α*) transcription and translation [23]. Expressed HIF‐1*α* protein regulates activity of enzymatic activity of the APP cleaving enzyme 1 (BACE1), a *β* secretase [23, 24]. Supporting the hypoxia role mediating A*β* production are findings showing that HIF‐1*α* also binds and activates *γ*‐secretase, employing an alternative mechanism in the amyloidogenic pathway [23–25]. Therefore, *β* and *γ*‐secretase facilitate the processing of APP and the generation of soluble APP C‐terminal fragment‐*β* (*β*CTF). Subsequently, *β* and *γ*‐secretase cleave the *β*CTF to generate fragments of various lengths, including A*β* 1‐40 and A*β* 1‐42 peptides [13, 23].

It has been shown in cultured cells that hypoxia favours the accumulation of A*β*_42_ by reducing the expression of the *α* secretase enzyme, desintegrin and metalloproteinase domain‐containing protein 10 (ADAM10) [26, 27]. ADAM10 is required to cleave APP within the A*β* section, forming large N‐terminal fragments (sAPP*α*). This nonamyloidogenic pathway prevents the formation of A*β* [28]. However, if ADAM10 and therefore sAPP*α* production is reduced, the cleaving of APP is shifted to the amyloidogenic pathway which favours *β* and *γ*‐secretases; this cleaves APP in an alternative location which releases the A*β* fragment.

Hypoxia has been associated with mitochondrial and oxidative stress which leads to inflammation and contributes to the hyperphosphorylation of tau [1, 29, 30]. Tau′s primary role is to stabilise the cytoskeleton of axons by binding to the microtubules. Protein kinases and phosphatases are responsible for maintaining the phosphorylation of tau levels to maintain a tight interaction of tau with the microtubules and providing axonal stability [31]. Kinases, such as extracellular receptor kinase and proline‐directed protein kinases, can be activated by oxidative stress and A*β* aggregates [29]. An imbalance of the protein kinases′ activity can cause hyperphosphorylation of tau protein, which loses its function in stabilising the microtubule in the axon cytoskeleton causing neuronal and synaptic loss in the brain [29].

Taking into consideration that hypoxia regulates the processing of APP and increases the production of A*β* fragments and increased cellular inflammation disrupts mitochondria function leading to tau hyperphosphorylation which contributes to the formation of neurofibrillary tangles, this study aims to investigate the relationship between the concentration levels of A*β*_42_ and phosphorylated tau at threonine 181 (p‐tau181) in the blood with hypoxia parameters in moderate to severe OSAHS. Concentration levels of total tau (T‐tau) and A*β*_40_ will also be assessed.

## 2. Method

### 2.1. Experimental Design and Study Selection

The review was registered with OSF (DOI: 10.17605/OSF.IO/JB3FP) and was performed in accordance with the Preferred Reporting Items for Systematic Review and Meta‐Analyses (PRISMA) 2020 guidelines [32].

A literature search was performed using three databases, with no time frame imposed. The search terms used for each database are listed in Table 1. The titles of the studies were screened by two authors independently (S.P. and S.W.) to discard irrelevant and duplicated studies and to simplify the following stages in the study selection process. To increase objectivity and reduce errors, the next step of the eligibility process for accepting or rejecting the research papers to be included in the systematic review was the revision of the abstract.

**Table 1:** Search terms used in each database.

Database	Search term
PubMed	(‘OSA’ OR ‘obstructive sleep apnoea’ OR ‘obstructive sleep apnea’ [title/abstract]) AND (‘amyloid beta’ OR ‘A*β*42/40’ OR ‘A*β*42’ OR ‘A*β*40’ OR ‘tau’ [title/abstract])
Scopus	(‘OSA’ OR ‘obstructive sleep apnoea’ OR ‘obstructive sleep apnea’ [title/abstract/keywords]) AND (‘amyloid beta’ OR ‘A*β*42/40’ OR ‘A*β*42’ OR ‘A*β*40’ OR ‘tau’ [title/abstract/keywords])
Web of Science	(‘OSA’ OR ‘obstructive sleep apnoea’ OR ‘obstructive sleep apnea’ [abstract]) AND (‘amyloid beta’ OR ‘A*β*42/40’ OR ‘A*β*42’ OR ‘A*β*40’ OR ‘tau’ [abstract])

Abbreviations: A*β*, amyloid beta; OSA, obstructive sleep apnoea.

Abstracts were reviewed independently by two authors (S.P. and S.A.L.C.), and if an abstract met the inclusion criteria, it was then included in the full article review. If no sufficient information was available on the abstract to apply the full criteria, the full text publication was assessed. A meeting to discuss the outcome of the selection process took place to consider the reasons for selecting and rejecting the studies. There was disagreement in two out of 38 papers. Both papers were reviewed, discussed and justifiable reasonings for the outcome was concluded and agreed by the two authors. The discrepancies included a misunderstanding regarding the inclusion of blood biomarkers and a control group due to ambiguity in the abstracts. Both papers were omitted from the meta‐analysis. Although availability of a third author to resolve any dispute was available, this process was not required. The references listed in the included papers were also screened by the authors.

### 2.2. Eligibility Criteria

To be considered for inclusion, studies were required to meet the specific inclusion criteria: (1) articles should be written in English; (2) have no restriction in publication time; (3) evaluate an experimental group diagnosed with OSAHS; (4) use of polysomnography for the diagnosis of OSAHS; (5) a comparator (control) group; (6) blood sample for the analysis of AD biomarkers including A*β*_40_, A*β*_42_, T‐tau, p‐tau217 and p‐tau181; (7) utilise a randomised control trial, cohort and case‐controlled studies; (8) studies that made the data available for extraction using means and SD (in the publication or by contacting the authors). The exclusion criteria were (1) subjective methods used to diagnose OSAHS; (2) animal models; (3) duplicate articles; and (4) use of non‐parametric statistical measures, i.e., median and interquartile range.

### 2.3. Quality Assessment of the Evidence

The quality of each paper was assessed using the Newcastle–Ottawa Scale (NOS) [33]. Each article was measured against the 7‐star scale to assess the selection, comparability and outcome. The interpretation used for the quality of the paper was as follows: higher scores indicated higher quality of paper, with a score of 0–2 indicating low quality, 3 and 5 indicating medium quality and ≥ 6 indicating high quality. Two authors (S.P. and S.A.L.C.) independently scored the articles, and any discrepancy in the score was discussed and resolved between the two authors.

### 2.4. Data Extraction

Data extraction was performed using a standard proforma in an Excel spreadsheet (Version 16.89.1). The following information was extracted from the included articles: (1) first author′s last name; (2) name of the journal or source; (3) publication year; (4) country; (5) study design; (6) sample size; (7) severity of OSAHS; (8) number of male and female participants; (9) mean age; (10) mean body mass index (BMI); (11) apnoea hypopnoea index (AHI); (12) oxygen desaturation index (ODI); (13) mean oxygen saturation (SpO_2_); and (14) serum or plasma, A*β*_40_, A*β*_42_, T‐tau, p‐tau217 and p‐tau181. In the longitudinal studies, only baseline data were extracted.

### 2.5. Data Analysis

To evaluate the concentration of AD biomarkers (A*β*_40_, A*β*_42_, T‐tau and p‐tau181), a meta‐analysis was performed using RevMan (Review Manager, Version 5.4). The standard mean difference was used to evaluate the differences between AD biomarker concentrations measured in the plasma or serum and the values of the hypoxic parameters. The degree of heterogeneity between studies was measured using *I*‐squared statistic, which measure the percentage of variation between studies. Heterogeneity was assessed using 25%, 50% and 75% corresponding to low, moderate and high heterogeneity. In all cases of heterogeneity, the random effects model was used to provide robust interpretation and generalisation of the results as opposed to the fixed effects model [34]. Continuous data reported as mean standard error and confidence intervals were converted to standard deviation using Cochrane methods [35]. Publication bias was assessed by constructing funnel plots and visual inspection. An asymmetrical funnel plot is indicative of small‐study effects which may suggest publication bias [36]. We were unable to quantify the level of publication bias such as the Egger′s test because of the small number of studies meeting the inclusion criteria of the meta‐analysis [36].

The subgroup analysis was conducted using mean SpO_2_ and ODI. The thresholds used were based on clinically accepted normal versus abnormal levels within each parameter. Therefore, the ODI threshold used was moderate (≥ 15 events per hour), and finally, the threshold for the mean SpO_2_ was 93% to represent a healthy saturation of ≥ 94% [37–39]. Previous studies have used a mean SpO_2_ threshold of 90%, though the reasoning for this is unclear. An oxygen saturation of ≤ 90% is suggestive of respiratory failure; however, hypoxaemia is defined at < 94% [40, 41]. Using the mean SpO_2_ 90% threshold will omit those with hypoxia between 94% and 90%. For example, a person with severe OSAHS (≥ 30 events per hour) will experience significant IH but maybe show a mean SpO_2_ of 92%. However, these individuals can still experience deep desaturations, resulting in significant IH throughout the night. The purpose of this study is to assess the impact of CIH on the plasma biomarkers; therefore, we used the threshold of clinical hypoxia at ≤ 93% to include those with a mean SpO_2_ between 93% and 91%.

## 3. Results

This meta‐analysis investigated the association between CIH and levels of AD protein biomarkers in the blood of moderate to severe OSAHS individuals compared with a control group. This study also examined whether increased hypoxic levels were associated with changes in blood AD biomarker concentrations in the OSAHS group.

### 3.1. Description of Included Studies

The initial phase of the literature search identified a total of 436 publications across three databases. With the use of the automation tools, 226 papers were removed because of an ineligibility to comply with the terms used to generate the filters. The terms used were full text studies; humans; adults only; and within the neurology, respiratory and sleep disciplines. Duplicate papers were removed which left 113 papers for title review. Out of 113 studies, 39 papers were included based on abstract review. One paper [20] was not retrieved as the data were presented in median and interquartile and therefore did not fulfil the inclusion criteria, which was to present the biomarker concentration values in pg/mL. We contacted the authors directly, but no response was received after a couple of attempts. After rigorous screening, applying the inclusion and exclusion criteria, eight papers met the inclusion criteria and hence the final number of studies included in the meta‐analysis [21, 42–48] (Figure 1).

**Figure 1:**
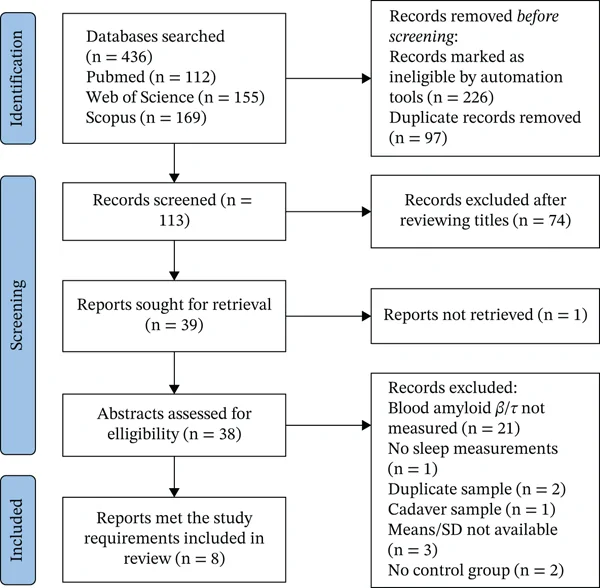
PRISMA flowchart showing the search strategy and study selection.

A total of 716 participants were included in the meta‐analysis, 435 were included in the OSAHS group and 281 were in the control group. Six research papers employed cross‐sectional design, and two studies were longitudinal. We have included the baseline findings for hypoxia and blood biomarker in the meta‐analysis. Seventy‐four percent of the participants were male (controls = 65*%*, OSAHS = 79*%*). The mean age of the participants ranged between 30.9 and 57.36 years old, and the BMI ranged between 23.02 and 35 (Table 2).

**Table 2:** Participant characteristics of the included studies.

Study	Year	Country	Study design	Sample size	Severity	Sex (m %)	Age (years)	BMI (kg/m^2^)
Bu et al. [21]	2015	China	CS	45	Mild, moderate and severe	73.33	44.31 ± 9.96	26.84 ± 2.52
				49	Controls	69.39	42.98 ± 9.62	26.07 ± 2.61
Motamedi et al. [42]	2018	USA	CS	22	Mild	95.5	34 ± 8.19	30.5 ± 4.8
				28	Moderate and severe	100	35.6 ± 7.77	31.1 ± 3.75
				24	Controls	91.7	30.9 ± 7.77	28.6 ± 3.81
Madaeva et al. [43]	2019	Russia	L	24	Moderate and severe	58.3	52.5 ± 2.7	30.2 ± 4.8
				20	Controls	60	49.1 ± 8.2	27.3 ± 1.5
Przybylska‐Kuć et al. [44]	2019	Poland	CS	38	Mild and moderate	54.8	52.2 ± 10.1	32.1 ± 6.8
				43	Severe	81.6	54.4 ± 10.4	35 ± 6.3
				31	Controls	86	46.1 ± 14.1	31.4 ± 6.1
Kong et al. [45]	2021	China	CS	35	Severe	85.8	39.18 ± 9.22	25.65 ± 2.78
				16	Controls	87.5	42.43 ± 9.79	23.02 ± 2.7
Chen et al. [46]	2022	Taiwan	CS	30	Mild, moderate and severe	90	41.93 ± 9.04	26.18 ± 2.85
				34	Controls	50	43.21 ± 13.12	24.83 ± 3.15
Li et al. [47]	2022	China	L	118	Mild, moderate and severe	72.03	57.36 ± 11.1	28.01 ± 4.19
				64	Controls	59.38	57.28 ± 9.18	25 ± 3.71
Sun et al. [48]	2022	China	CS	52	Mild, moderate and severe	73	53.81 ± 7.04	27.59 ± 4.58
				43	Controls	70	54.61 ± 7.28	26.23 ± 4.16

*Note:* Data presented as mean (± SD).

Abbreviations: BMI, body mass index; CS, cross‐sectional, L: longitudinal; NR, not recorded.

In the OSAHS group, the mean AHI ranged between 15.7 and 63.92 per hour, ODI ranged between 20.2 and 64.57 per hour, and mean SpO_2_ ranged between 80.4% and 95%. Six studies analysed plasma (four used the ELISA assay, one used the Simoa assay and one used IMR assay), and two studies analysed serum using the ELISA assay (Table 3). There were not sufficient papers that met the inclusion criteria and provided the A*β*_42_/A*β*_40_ ratio and p‐tau217 concentration levels in OSAHS; and they therefore could not be included in this meta‐analysis.

**Table 3:** Extracted and summarised details of hypoxic parameters and AD biomarkers in OSAHS and control groups.

Study	Sample type	Severity	A*β*_40_ (pg/mL)	A*β*_42_ (pg/mL)	T‐tau (pg/mL)	p‐tau 181 (pg/mL)	AHI (/hr)	SpO_2_ (%)	ODI (/hr)
Bu et al., 2015	Serum—ELISA	Mild, moderate and severe	107.54 ± 65.77	86.37 ± 61.14	45.4 ± 34.24	42.03 ± 21.58	32.68 ± 23.34	90.93 ± 2.90	26.45 ± 17.76
		Controls	71.58 ± 34.7	50.91 ± 26.93	40.49 ± 24.61	30.56 ± 17.3	2.26 ± 1.35	95.61 ± 1.17	2.17 ± 1.27
Motamedi et al., 2018	Plasma—Simoa	Mild	NR	NR	2.95 ± 2.02	NR	8.56 ± 2.5	NR	NR
		Moderate and severe	NR	NR	5.39 ± 2.57	NR	26.09 ± 21.3	NR	NR
		Controls	NR	NR	2.48 ± 1.94	NR	2.12 ± 1.3	NR	NR
Madaeva et al., 2019	Plasma—ELISA	Moderate and severe	NR	2.25 ± 3.91	NR	NR	38.6 ± 18.2	80.4 ± 5.10	NR
		Controls	NR	4.5 ± 6.62	NR	NR	3.89 ± 1.35	96.7 ± 1.2	NR
Przybylska‐Kuć et al., 2019	Serum—ELISA	Mild and moderate	NR	85.9 ± 60.1	NR	NR	15.7 ± 7.2	92.2 ± 1.90	20.2 ± 14.5
		Severe	NR	86.9 ± 53.3	NR	NR	54.1 ± 17.7	88.9 ± 3.90	59.5 ± 20.7
		Controls	NR	79.5 ± 54.1	NR	NR	2 ± 1.7	92.6 ± 1.80	5.3 ± 6.1
Kong et al., 2021	Plasma—ELISA	Severe	29.24 ± 32.52	0.23 ± 0.17	11.88 ± 7.05	26.31 ± 14.41	63.92 ± 23.65	91.43 ± 3.40	64.57 ± 26.82
		Controls	13.18 ± 10.78	0.26 ± 0.22	7.64 ± 4.17	17.34 ± 9.12	2.62 ± 1.93	95.29 ± 0.99	2.81 ± 2.25
Chen et al., 2022	Plasma—IMR	Mild, moderate and severe	53 ± 4.93	16.17 ± 0.66	21.43 ± 2.85	NR	41.93 ± 23.72	95 ± 1.48	32.75 ± 23.11
		Controls	54.61 ± 7.29	15.37 ± 1.52	18.27 ± 3.15	NR	2.68 ± 1.69	97.06 ± 1.11	0.91 ± 1.22
Li et al., 2022	Plasma—ELISA	Mild, moderate and severe	NR	2.98 ± 0.71	NR	NR	NR	NR	NR
		Controls	NR	2.76 ± 0.65	NR	NR	NR	NR	NR
Sun et al., 2022	Plasma—ELISA	Mild, moderate and severe	NR	2.95 ± 0.97	153.65 ± 34.22	41.42 ± 12.53	34.8 ± 20.45	92.73 ± 2.13	29.45 ± 16.99
		Controls	NR	2.56 ± 0.76	142.47 ± 12.53	40.53 ± 11.28	2.81 ± 1.18	95.4 ± 1.43	2.19 ± 1.22

*Note:* Data presented as mean (±SD).

Abbreviations: AHI, apnoea and hypopnoea index; A*β*, amyloid beta; IMR, immunomagnetic reduction; ODI, oxygen desaturation index; p‐tau, phosphorylated tau; SpO_2_, oxygen saturation; T‐tau, total tau.

All papers were evaluated using the NOS assessment for cross‐sectional studies. This allowed each paper to be assessed for quality based on three overarching categories: (1) three stars score (representativeness of the sample: was the cohort confirmed with OSAHS, were there evidence of non‐respondents participants and was evidence of validated tool used for diagnosis, e.g., polysomnography to ensure OSAHS was accurately measured); (2) two stars score (inclusion of controls for most important factor, e.g., BMI and for additional factor, e.g., sex); and (3) outcome maximum of two stars (assessment of outcome was performed blind, data reported and analysed using statistical tests, and the measured parameters were described and appropriately used in the study). Six studies were of medium quality, and two studies were of high quality according to NOS (Table 4). There were no studies of low quality included in the meta‐analysis.

**Table 4:** Quality assessment of included studies using the Newcastle–Ottawa scale score.

Study	Selection			Comparability		Outcomes		Total
Author	Sample represe‐ntation	Non‐respondents	Ascertainment of exposure	Primary study control	Additional study controls	Assessment	Statistical test	
Bu et al., 2015	★	—	★	★	★	—	★	5^★^
Motamedi et al., 2018	★	—	★	★	★	—	★	5^★^
Madaeva et al., 2019	★	—	★	★	★	—	★	5^★^
Przybylska‐Kuć et al., 2019	★	—	★	★	★	—	★	5^★^
Kong et al., 2021	★	—	★	★	★	—	★	5^★^
Chen et al., 2022	★	—	★	★	★	★	★	6^★^
Li et al., 2022	★	—	★	★	—	★	★	5^★^
Sun et al., 2022	★	—	★	★	★	★	★	6^★^

### 3.2. Pooled Analysis

To investigate whether the concentration of blood biomarkers (A*β*_42_, A*β*_40_, T‐tau and p‐tau181) were altered in OSAHS, we compared levels of each biomarker between OSAHS and control groups. Out of the eight studies included in the meta‐analysis, seven reported concentrations of A*β*_42_ (*n* = 385 OSAHS and *n* = 257 controls) [21, 43–48]. Participants with OSAHS had significantly higher concentration of A*β*_42_ in the blood when compared with controls (SMD = 0.27, 95% (CI) = 0.03–0.51, effect size = 2.18 [*p* = 0.03]) (Figure 2A). Please note that the total number of controls in Figure 2A is 288, which is a higher number than the total controls included in this study. This is because two experimental groups from the Przybylska et al. study [44] (mild and moderate OSAHS and severe OSAHS), which was controlled by one group of participants, was included in the analysis. Therefore, to allow for a comparison across the two experimental groups, this study was inputted twice into the RevMan software, which automatically displayed the total number of controls in Figure 2A as 288 and not 257. A*β*_40_ concentration was analysed in three studies (*n* = 110 OSAHS and *n* = 99 controls) showing no significant difference between groups (SMD = 0.33, 95% (CI) = −0.27 to 0.94, effect size = 1.09 [*p* = 0.28]) (Figure 2B) [21, 45, 46]. A significant increase in T‐tau concentration was observed in OSAHS participants compared with controls in five studies (*n* = 212 OSAHS and *n* = 190 controls) [21, 42, 45, 46, 48] (SMD = 0.60, 95% (CI) = 0.26–0.94, effect size = 3.42 [*p* = 0.0006]) (Figure 2C). Similar findings were observed for p‐tau181 in three studies (*n* = 132 OSAHS and *n* = 108 controls) [21, 45, 48] (SMD = 0.41, 95% (CI) = 0.03–0.80, effect size = 2.12 [*p* = 0.03]) (Figure 2D).

**Figure 2:**
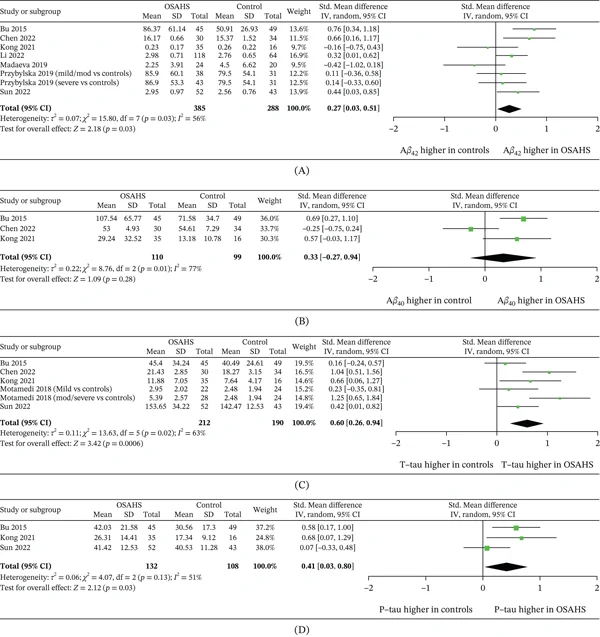
Forest plot comparing the Alzheimer′s disease (AD) blood biomarker levels in participants with OSAHS and controls: (A) A*β*_42_; (B) A*β*_40_; (C) T‐tau; (D) p‐tau181.

We calculated the A*β*_42_/A*β*_40_ ratio in three studies (*n* = 110 OSAHS and *n* = 99 controls) [21, 45, 46] showing no significant difference between groups (SMD = 0.16, 95% (CI) = −0.20 to 0.51, effect size = 0.87 [*p* = 0.38]) (Figure 3).

**Figure 3:**
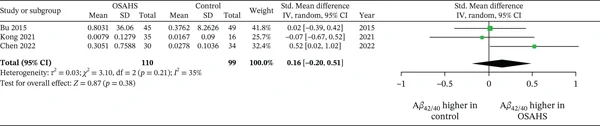
Forest plot comparing the A*β*_42_/A*β*_40_ ratio blood biomarker levels in participants with OSAHS and controls.

### 3.3. Sensitivity Analysis

As the heterogeneity was elevated for the A*β*_42_, A*β*_40_, T‐tau and p‐tau181, sensitivity analysis was performed. Sensitivity analysis showed that the A*β*_42_ and T‐tau results have not significantly changed when sequentially removing each study, confirming the robustness of the results. However, A*β*_40_ results showed improved heterogeneity after removal of the Chen et al. study [46] (SMD = 0.33, 95% (CI) = −0.27 to 0.94, effect size = 1.09, *I* ^2^ = 77*%*, *p* = 0.28 vs. SMD = 0.65, 95% (CI) = 0.31–0.99, effect size = 3.71, *I* ^2^ = 0*%*, *p* = 0.0002) (Figure 4). This demonstrated an increased association with the OSAHS group.

**Figure 4:**
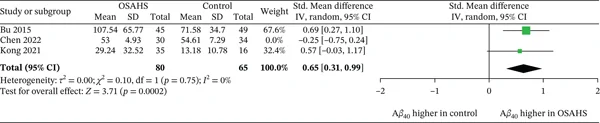
Forest plot showing reduction in heterogeneity for A*β*_40_ analyses after removing Chen et al. [46].

In addition, p‐tau181 results showed improved heterogeneity after removal of the Sun et al. [48] study (SMD = 0.41, 95% (CI) = 0.03–0.80, effect size = 2.12, *I* ^2^ = 51*%*, *p* = 0.03 vs. SMD = 0.61, 95% (CI) = 0.27–0.96, effect size = 3.52, *I* ^2^ = 0*%*, *p* = 0.0004) (Figure 5). This demonstrated an increased association with the OSAHS group.

**Figure 5:**
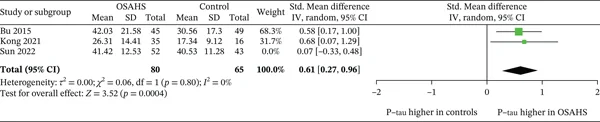
Forest plot showing improved heterogeneity in p‐tau181 comparisons after excluding p‐tau181 measurements from Sun et al. [48].

### 3.4. Subgroup Analysis

To evaluate whether hypoxic parameters and OSAHS severity were associated with concentration of AD biomarkers, subgroups were stratified by cut off values: mean SpO_2_ (≤/> 93%) and ODI (≥ 15 events per hour).

### 3.5. Mean Oxygen Saturation and ODI

Hypoxic condition has been described to facilitate APP processing and inhibit the downregulation of A*β* peptides, including A*β*_40_ and A*β*_42_ [23–27]. We therefore hypothesised that increased levels of CIH observed in OSAHS are associated with increased concentration levels of A*β*_40_ and A*β*_42_ and therefore hypoxia may be a contributing factor for increasing levels of A*β* peptides in the blood.

Findings for four studies were included to investigate the association between levels of blood biomarkers and mean SpO_2_ [21, 43, 45, 48]. Analyses showed no significant correlation in levels of A*β*_42_ between groups that experience SpO_2_ of ≤ 93% and a normal SpO_2_ of > 93% (SMD = −0.19, 95% (CI) = −0.70 to 0.32, effect size = 0.75 [*p* = 0.46]) (Figure 6A). Interestingly, a significant association between increased levels of T‐tau (SMD = −0.36, 95*%*(CI) = −0.62 to − 0.10, effect size = 2.69 [*p* = 0.007]) (Figure 6B) and p‐tau181 (SMD = −0.61, 95*%*(CI) = −0.96 for − 0.27, effect size = 3.52 [*p* = 0.0004]) (Figure 6C) was found in participants with a mean SpO_2_ of ≤ 93% compared with participants that demonstrated a mean SpO_2_ of > 93%. Blood biomarkers A*β*_40_ were not analysed in relation to the mean SpO_2_ because of an insufficient number of studies. These findings show that a reduced average oxygen level below 93% may not be a suitable parameter to assess production and/or clearance of A*β*.

**Figure 6:**
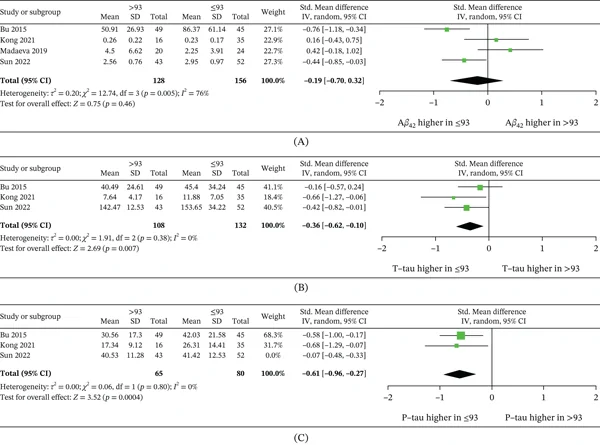
Forest plot comparing the mean oxygen saturation with blood biomarkers: (A) A*β*_42_; (B) T‐tau; (C) p‐tau181.

We then investigated whether the number of times that the oxygen levels in the blood dropped by more than 4% from the baseline per hour during sleep, causing CIH, would affect brain function. Out of the eight studies included, five measured AD biomarkers and ODI [21, 44–46, 48]. A significant correlation was found between increased levels of A*β*_42_ (SMD = −0.37, 95*%*(CI) = −0.56 to − 0.18, effect size = 3.80 [*p* = 0.0001]) (Figure 7A) and T‐tau (SMD = −0.54, 95*%*(CI) = −0.90 to − 0.17, effect size = 2.87 [*p* = 0.004]) (Figure 7B) in participants with an ODI of ≥ 15 events per hour compared with participants that experienced an ODI of < 15 events per hour. As these studies did not measure levels of A*β*_40_ and p‐tau181, it was not possible to determine the correlations between ODI and A*β*_40_ concentrations.

**Figure 7:**
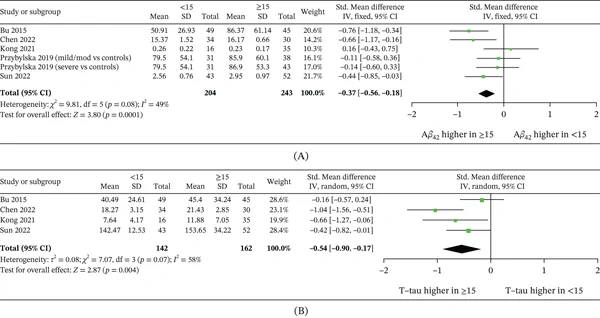
Forest plot comparing the oxygen desaturation index with AD blood biomarkers: (A) A*β*_42_; (B) T‐tau.

### 3.6. Publication Bias

Visual inspection of funnel plots suggests an asymmetrical pattern and therefore evidence of publication bias for the evaluation of biomarkers A*β*_42_ and T‐tau in the OSAHS group compared with controls (Figure 8A,B). We recognised that funnel plots should be interpreted with caution because of the limited number of papers included in the meta‐analysis. Likewise, we were unable to assess publication bias in A*β*_40_ and p‐tau181 because of each only having three studies.

**Figure 8:**
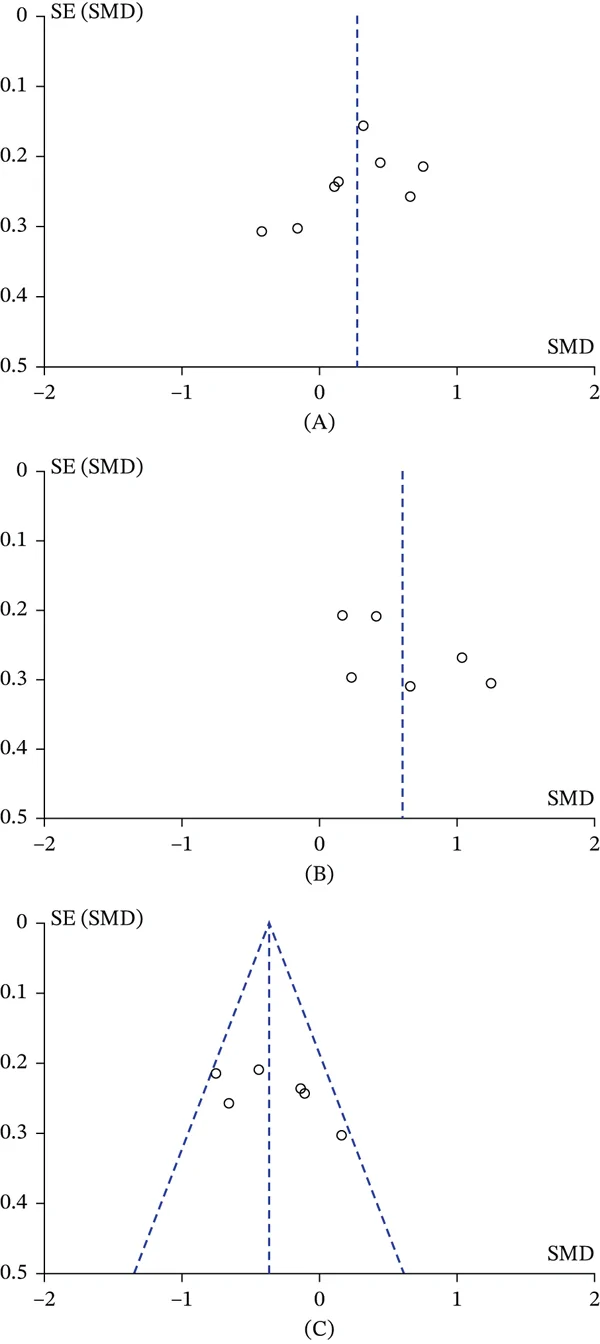
Funnel plots assessing publication bias: (A) OSAHS, controls and A*β*_42_; (B) OSAHS, controls and T‐tau; (C) ODI and A*β*_42_.

There were few studies that reported mean SpO_2_ to allow for the assessment of publication bias. There was, however, evidence of publication bias in the ODI subgroup studies (Figure 6C), and therefore, this finding should be cautiously interpreted because of the low number of studies. Publication bias was not assessed in the T‐tau and ODI group because of few papers available.

## 4. Discussion

For this study, we employed a systematic review combined with meta‐analysis to investigate the relationship between levels of AD biomarkers in the blood of persons with OSAHS compared with controls and whether parameters associated with CIH correlated with changes in AD blood biomarkers in OSAHS. Analyses of findings from eight research studies that met the inclusion and exclusion criteria showed a significant increased concentration of A*β*_42_, T‐tau and p‐tau181 in the OSAHS group compared with the control group (Figure 2). In contrast, no significant difference was observed in levels of A*β*_40_ and A*β*_42_/A*β*_40_ ratio between groups.

Surprisingly, lower levels of mean SpO_2_ correlated with elevated levels of T‐tau and p‐tau181 but not with changes in A*β*_42_ levels (Figure 6A–C). Interestingly, higher frequency of oxygen drops in the blood was significantly associated with higher levels of A*β*_42_ and T‐tau in the blood (Figure 7A,B) of those with moderate and severe ODI compared with normal and mild ODI levels.

### 4.1. Association Between OSAHS and AD Blood Biomarkers and Implications

Several studies reported elevated levels of AD biomarkers in the blood of OSAHS individuals including two recent published systematic reviews and meta‐analyses [20, 22]. In agreement with [20–22, 48] studies, we found a significant increase in A*β*_42_ concentration in the OSAHS group when compared with controls, but this increase was not found in [43, 44] studies. In contrast to findings for these studies, we did not find a difference in the A*β*_40_ concentration in the blood of OSAHS group compared with controls. Our findings also contrast with a meta‐analysis which showed an increase in A*β*_40_ but not in A*β*_42_ concentrations in the blood [22, 48, 49].

There are several reasons that may explain the different results between the studies. The current meta‐analysis included an additional study by Chen et al. [46] that was not published at the time the Kang et al. [49] meta‐analysis was performed. Although Chen et al. [46] was already published at the time that the Yeo et al. [22] meta‐analysis was performed, these authors also did not include this study in their publication. Based on the inclusion and exclusion criteria used in Yeo et al. [22] study, it is not clear why the authors have not included the Chen et al. [46] study. In addition, the Bhuniya et al., 2022 [20] study included in the Yeo et al. [22] meta‐analysis was omitted from our analysis due to the use of non‐parametric metrics (median and interquartile range) rather than parametric metrics (mean and standard deviation). The inclusion of parametric and non‐parametric results may induce bias depending on the skewness of the data reducing the statistical rigour in Yeo et al. [22] study. This may cause misinterpretations of the dataset and the association between OSAHS and AD blood biomarkers.

Another potential factor for the discrepancy between studies is the use of different protocols to prepare the blood samples and technologies such as ELISA, Simoa and immunomagnetic reduction (IMR) to measure the concentration of biomarkers. Differences in detection sensitivity and accuracy are likely to contribute to inconsistencies in the results.

An important point to consider for the interpretation of these results is the inconsistency in blood A*β*_42_ concentrations reported from independent studies. For example, Chiu et al. [50] reported an increase in A*β*_42_ but no changes in A*β*_40_ concentrations in mild to severe AD compared with controls. These experiments were performed using an ultrasensitive immunoassay, named IMR assay, developed to quantify blood A*β*_42_ in the plasma. To assess the correlation between expression levels of A*β*_42_ in the plasma and CSF, Teunissen et al. [51] employed IMR assay to measure plasma biomarkers and commercially ELISA (Innotest *β*‐amyloid [1‐42]; Fujirebio) to quantify A*β*_42_ levels in the CSF in the same cohort [51]. This study found that A*β*_42_ levels in the plasma are negatively correlated with values detected in the CSF of AD cohort when compared with controls. A similar study showed a significant reduction in A*β*_42_ and A*β*_40_ concentrations in the plasma of AD when compared with cognitively impaired and control groups employing the Simoa immunoassay (Quanterix) to measure A*β* levels in the plasma and Euroimmun immunoassay to assess CSF [52]. Significant reductions in CSF A*β*_42_ levels were observed in AD group compared with controls and cognitively impaired groups, but no difference was observed in A*β*_40_ concentration between AD and controls. These findings illustrate the challenges in interpreting results from different laboratories using different technologies. These findings also illustrate the importance of evaluating the accuracy and sensitivity of the current technology in clinical settings.

Supporting the presence of altered AD‐related biomarkers in OSAHS is the significant increase in p‐tau181 found in the OSAHS group compared with controls. It has been recognised that elevated levels of p‐tau181 in the plasma are accurate to predict cerebral deposition of A*β*, tau pathologies, and it can be used as a predictor of cognitive decline [52]. In the revised criteria for the diagnosis of AD, p‐tau181 has been included as core fluid biomarker as well as phosphorylated tau at threonine 217 (p‐tau217) and 231 (p‐tau231) residues [16]. Recent evidence shows that altered p‐tau217 concentration in the plasma has shown similar accuracy to CSF biomarkers for detecting A*β* and tau pathologies in symptomatic patients [53]. Furthermore, longitudinal changes in plasma p‐tau217 concentrations have been proposed to predict the length of time for cognitively intact individuals to develop the AD clinical symptoms [54]. AD pathology progression has been associated with changes in fluid AD biomarker levels in adults with Down syndrome (DS), who are at high risk of developing AD dementia [55]. A cross‐sectional study measured A*β*_42_ concentration in the plasma of DS persons at asymptomatic, prodromal and AD dementia pathological stages when compared with aged cognitively healthy euploid matched controls [56]. A significant increase in concentration of A*β*_42_ plasma was observed in DS samples compared with controls, but this increase has not changed with AD pathology progression seen in DS adults. However, there was a significant step increase in plasma p‐tau181 concentrations from asymptomatic to AD dementia stages in DS samples when compared with controls [57]. These findings suggest that longitudinal change in p‐tau181 concentration is a more specific biomarker compared with A*β*_42_ to identify AD pathological progression. Our findings of elevated p‐tau181 in the blood of OSAHS group suggest the presence of biological changes that are associated with AD.

OSAHS is a multifactorial disease, and those with OSAHS are more likely to possess comorbidities such as diabetes and hypertension which are recognised modifiable risk factors to the development of AD [9, 58–60]. For example, hypertension is a major modifiable risk factor for AD development; sustained high blood pressure reduces brain artery diameter and their stiffness which reduce flow and oxygen delivery to the brain [61]. This causes hypoxia which may lead to increased production of, for example, A*β*_42_. Reduction in arterial elasticity disrupts fluid flow in and out of the brain parenchyma affecting the glymphatic system function and clearance of waste proteins from the brain [62, 63]. These changes are likely to contribute to changes in the brain of OSAHS sufferers and consequently contribute to increased level of Ad‐related biomarkers observed in these individuals. Another example is that high levels of glucose observed in diabetes can cause damage to vessels, increase inflammation and trigger microglial activation which reduces clearance of A*β* from the brain [64]. Although it is not known which comorbidity may appear first in OSAHS sufferers, it is known that IH and hence unstable oxygen levels in the blood cause blood pressure swings during the night. This may contribute to the development of hypertension in OSAHS individuals [17]. In addition, OSAHS may increase cortisol levels in the blood which can alter glucose regulation, and those with OSAHS are more likely to be obese, another risk factor for diabetes [59]. Chronic elevated levels of cortisol can contribute to glucose intolerance and insulin resistance [59]. Both examples of confounders are intricately linked to OSAHS and known modifiable AD risk factors. As well as modifiable risk factors, another consideration is the impact of natural ageing increasing the risk of developing AD [9]. However, age may not be a critical factor for interpretation of findings in this study as the mean age of individuals included in this study is 30.9 and 57.36 years old for control and OSAHS, respectively.

The significance of the physiological changes associated with OSAHS increases with the disease severity is likely to influence the biomaker distribution profile. The inclusion of mild, moderate and severe OSAHS as one group in our study is most likely a contributing factor to the skewed data by reducing the statistical power and the relevance of the findings. Most of the included studies combined mild, moderate and severe biomarker concentrations, and therefore, this study was unable to separate the distribution of data across the OSAHS severity groups. However, one study, Motamedi et al. [42] demonstrated a larger T‐tau concentration level in the moderate to severe OSAHS group (5.39 ± 2.57 pg/mL) compared with the mild OSAHS group (2.95 ± 2.02 pg/mL). This suggests that the use of a mild OSAHS group may generate an increased variance in the dataset. Several studies such as Bu et al. (2015) [21] demonstrated very large standard deviations suggesting large variability or outliers in the study cohorts. This may reduce the true representation of the mean results from the individual studies, skewing the overall combined mean. Several studies crossed the vertical line of no effect in the forest plot, suggesting no individual significance. Although caution should be employed with the interpretation of the data, it is important to highlight the progress made and areas to be considered in the design of future research in this area.

The impact of other comorbidities such as APOE genotype, cardiovascular disease, diabetes, hypertension and renal function was not assessed in the studies included in this study; therefore, we are unable to speculate about the contribution of these confounders affecting the concentration levels of AD biomarkers described in this study or whether they contributed to high standard deviations observed in this dataset. Therefore, with the data available at the time this study was performed, the generalisation of the elevated plasma AD biomarkers directly associated with OSAHS is limited.

### 4.2. Association Between Hypoxic Parameters (SpO_2_ and ODI) and Blood Biomarkers and Its Implications

Our findings support the proposed hypothesis that CIH is associated with increased concentrations of A*β*_42_, T‐tau and p‐tau181 in blood. However, further experimental research is required to demonstrate whether an increase A*β*_42_ concentration levels results from a hypoxia‐mediated increase in the proteolysis of APP using the amyloidosis pathway in the OSAHS group compared with controls. In addition, the production and accumulation of A*β* contribute to the activation of signalling pathways that cause the hyperphosphorylation of tau resulting in increases in p‐tau181 concentrations in the brain, CSF and blood [29].

Reduced levels of A*β*_42_ concentration have been reported in the CSF of individuals with OSAHS [18, 65]. This suggests that A*β*_42_ peptides accumulate in the brain and facilitate the formation of A*β* plaques [65]. However, often discrepancies are reported in the A*β*_42_ levels found in CSF and blood, which may be explained by samples being collected at different stages of AD. AD‐associated biomarkers begin to accumulate many years prior to the presentation of clinical symptoms [16, 66]. However, individuals are likely to have their CSF samples collected for analyses of biomarkers to confirm diagnosis of AD when cognitive impairment and the clinical symptoms of the disease are apparent. Therefore, the development of plasma technology to measure AD biomarker concentration levels (p‐tau217 and brain‐derived p‐tau217) may offer the opportunity for earlier detection of the AD biology before the clinical symptoms are apparent [67, 68]. In the future, it would be important to measure p‐tau217 levels in the plasma of newly diagnosed OSAHS persons. If the test is confirmed, these findings would predict whether OSAHS patients are at risk of developing AD. Screening the population for OSAHS and AD biomarkers would be an efficient method to treat OSAHS early on and perhaps to mitigate some of the risks associated with AD progression to cognitive impairment.

Findings from our study showed a significant correlation between increased ODI and A*β*_42_ concentration in the blood. One possible explanation for increases in A*β*_42_ concentration is a change in the mechanism regulating APP processing. Indeed, APP processing via the amyloidogenic pathway using *β* and *γ*‐secretase is activated, and the nonamyloidogenic pathway may be downregulated in cells exposed to hypoxia [24, 27]. This is supported by an experimental study that demonstrated a positive correlation between the production of A*β*_42_ and BACE1 when exposed to hypoxia [23].

Increases in T‐tau and p‐tau181 were associated with a reduction in the mean SpO_2_ when a low threshold of SpO_2_was set at ≤ 93%, whereas there was not a significant association with A*β*_42_ and mean SpO_2_. As p‐tau181 is a specific protein found predominantly in the axons of neurones, the increase found may represent an increase of tau in the brain [29]. This supports the findings from several studies that have demonstrated increased p‐tau181 blood levels being elevated in AD [69–72]. There has also been an interest in the timing of the tau progression in the brain with consideration of a ‘tau‐first’ variant, in which neurofibrillary tangles are widespread prior to the widespread development of A*β* [73]. This may suggest that the ‘tau first’ variant was more prevalent in this cohort, highlighting the importance of measuring tau in the diagnosis of AD.

In addition, the results of this meta‐analysis found a correlation between hypoxia and elevated tau levels in the blood. This suggests that hypoxia may influence the accumulation of p‐tau181 proteins in the brain and blood, and increasing concentration levels of p‐tau181 are associated neuronal injury [21, 74]. Furthermore, hypoxia leads to damaged endothelial cells, increased inflammation and increased oxidative stress, also contributing to neuronal injury [74]. This suggests that hypoxia may contribute to neuronal damage and the development of AD biomarkers in OSAHS patients. However, other OSAHS‐dependent physiological changes including those observed in sleep architecture, including AHI and amount of slow‐wave sleep, may contribute to changes in AD biomarker concentrations in the plasma [44, 75].

### 4.3. Clinical Significance of Findings

The effect sizes observed in this meta‐analysis were small and would not be of clinical significance. This reflects the limited number of studies that meet the defined inclusion criteria for this study. The high heterogeneity of the studies included was another contributing factor to the low statistical impact of these findings. More studies employing robust and rigorous experimental design are required to determine whether OSAHS is associated with changes in AD biomarkers in the plasma.

Another important factor to determine the clinical significance of plasma AD biomarkers is to evaluate the sensitivity and accuracy of the immunoassays to detect and discriminate disease in the real‐world population. This information is required to determine the effect size of future studies and consequently their clinical utility. Progress has been made in evaluating immunoassays to measure AD biomarkers in the blood, including results showing that measurements p‐tau217 in the plasma using distinct immunoassays reliably identified amyloid‐PET, tau‐PET and cognitive abnormalities in a cohort composed of cognitively impaired and unimpaired individuals [52]. Although some immunoassays perform better than others in terms of sensitivity and accuracy, these findings are encouraging and support the utility of p‐tau217 in clinical practices. A potential issue with immunoassay technologies is their low detection sensitivity compared with mass spectrometry [52]. However, the development of immunoassay strategies to detect brain‐derived p‐tau217 in the blood may take this field further and make blood tests available to the wide population.

### 4.4. Considerations of the Included Studies

The age range of the OSAHS participants across the included studies was 34–57 years old which may have influenced the level of the biomarkers in the blood. Typically, sporadic AD is more common to occur in those over the age of 60 [7]. Despite this younger age group, changes to the brain including the deposition A*β* and neurofibrillary tangles of tau can appear several years before the onset of clinical symptoms (such as cognitive impairments) in AD [76]. Furthermore, those with OSAHS may transition to prodromal AD at a younger age compared with those without a sleep breathing disorder [49, 77]. This may explain the elevated A*β*_42_, p‐tau181 and T‐tau levels found in the OSAHS group in the current meta‐analysis. This is also supported by a meta‐analysis which found elevated blood A*β*_42_ levels in those with initial stages of AD [78]. The varying levels of the AD biomarkers previously reported may indicate a lower A*β*_42_ burden in the OSAHS brain and may be responsible for the transition to prodromal AD. This further supports the need to understand the impact of biomarkers at different disease trajectories.

Two studies included in this meta‐analysis focused on neural‐derived plasma exosomes (NDE) [47, 48]. Interestingly, both studies found NDE A*β*_42_ to be higher in OASHS and even higher in those with OSAHS and cognitive impairment, suggesting pathological changes are present [47, 48]. This indicates OSAHS influences the production of NDE A*β*_42_. As OSAHS patients suffer from severe sleep fragmentation and hypoxia, it is possible that clearance of A*β*_42_ from the brain through the glymphatic system is reduced [18]. In addition, increased levels of T‐tau and p‐tau181, which can contribute to neuronal injury, were found in OSAHS blood, and again, more so in those with OSAHS and cognitive impairment [48]. Consequently, both studies support an increased production of AD biomarkers specifically derived in the OSAHS brain.

The result of this meta‐analysis highlights the lack of standardisation in methodology to consistently measure the concentration levels of AD biomarkers in the blood. A current review of the guidelines to diagnose AD [16] has accepted that methodology to measure phosphorylated tau at amino acids 181, 217 and 232 is accurate and can be used in combination with amyloid‐PET to diagnose early stage of AD pathology before cognitive impairment is apparent. As illustrated in this meta‐analysis, the included studies for analyses used different assays and measurement thresholds, which likely affected the sensitivity, accuracy and reproducibility of measuring proteins in the blood. Despite different protocols, the findings from this study showed significant increased levels of p‐tau181 in the OSAHS group. As p‐tau181, 217 and 231 correlate well with the accumulation of amyloid‐*β* deposition in the brain, our findings showing significant increases in p‐tau181 plasma concentration suggest a biological change in the OSAHS brain that is consistent with AD pathology.

## 5. Limitations

There were several limitations to this meta‐analysis that need to be considered. First, most of the included studies used small sample sizes and were of a cross‐sectional design which will influence the reliability of the results. Second, the limited number of studies prevented rigorous analysis of publication bias, which may have overestimated the results. Third, the included studies were heavily biased towards the inclusion of male subjects, and finally grouping mild, severe and moderate OSAHS severity levels may have influenced biomarker distribution profile, increased standard deviation and data heterogeneity. Although OSAHS is more prevalent in males, AD is more prevalent in females, so demonstrating the association of the AD biomarkers in females with OSAHS is needed [8]. We also cannot assess whether other confounders were not influencing the outcome of the results such as obesity, cardiovascular disease, diabetes, APOE status, ethnicity and undetermined cognitive status because those with OSAHS are more likely to possess these confounding risk factors. Residual confounding is also a concern that may bias the results. Exclusion of non‐English published studies may have contributed to publication bias, particularly when using platforms like Web of Science and Scopus. However, studies have suggested that language restrictions to English‐only publications are unlikely to alter the conclusions of systematic reviews in conventional medicine using these platforms [79, 80]. Another limitation of this study is comparing levels of biomarkers in plasma and serum, which can affect the reproducibility of results. The use of different types of immunoassay and machines can also affect the measurements. However, these technologies have been extensively tested and approved for qualitative research use. Finally, the included studies were predominantly from Asian and Eastern populations which may not be fully translational to the Western population because of cultural, environmental and healthcare differences.

These limitations varied widely across the included studies, resulting in increased heterogeneity when comparing AD biomarker concentration levels between the control and OSAHS cohorts. Sensitivity analysis demonstrated improved heterogeneity in the A*β*_40_ and p‐tau biomarker comparisons. Interestingly, the A*β*_40_ sensitivity analysis improved when Chen et al. [46] was removed (Figure 4). This study had the highest mean SpO_2_ at 95% in the OSAHS cohort, whereas the other studies included in this analysis demonstrated mean SpO_2_ at 90.93% [21] and 91.43% [45]. In addition, Bu et al. (2015) [21] and Kong et al. [45] used the ELISA analytical platform, whereas Chen et al. [46] used IMR platform. The sensitivity analysis for p‐tau demonstrated heterogeneity improvement after the removal of Sun et al. [48]. They demonstrated similar sex and BMI distributions and analytical platform, yet age varied by 9.5 and 14.6 years when compared with Bu et al. [21] and Kong et al. [45], respectively. This demonstrates the increased variance across the studies associated with OSAHS severities, the methodology of the protein concentration levels, and the patient demographics, such as the age, are likely to have contributed to the large variance in the data from the included studies and the combined dataset used in this study.

## 6. Conclusion

This meta‐analysis has demonstrated a modest association between IH and elevated blood AD biomarkers in the OSAHS group. Specifically, an increased level of A*β*_42_ and p‐tau181 correlated with increased OSAHS severity, though causality cannot be determined. It has also been demonstrated that those with CIH were associated with elevated levels of p‐tau181, which may be associated with AD‐related pathology progression. Therefore, increased AD biomarkers in the OSAHS cohort are a biological change that may progress to pathological levels seen in early stages of AD. Consequently, if the OSAHS disorder and subsequently the IH are treated, this may modify the trajectory changes of the AD biomarkers. This supports OSAHS potentially being a modifiable risk factor of AD. However, there were methodological issues concerning confounding factors and heterogeneity across the included studies which may increase bias and skew the associated outcomes between the blood AD biomarkers and OSAHS. Therefore, more research employing longitudinal studies to assess a combination of AD biomarkers including the brain‐derived p‐tau217 is needed.

## Author Contributions

Sara Parsons: Conceptualisation; investigation; methodology; formal analysis; visualisation; writing—original draft preparation. Sam Williamson: Investigation; visualisation; methodology; writing—review and editing. Sonia A. L. Corrêa: Conceptualisation; investigation; supervision; visualisation; writing, revision, and editing.

## Funding

No funding was received for this manuscript.

## Disclosure

All authors have seen and approved the manuscript.

## Conflicts of Interest

The authors declare no conflicts of interest.

## Data Availability

The data that support the findings of this study are openly available in OSF registry at 10.17605/OSF.IO/JB3FP.
